# Development and Preliminary Verification of Specific KASP Markers for Mango Resistance to Bacterial Angular Leaf Spot

**DOI:** 10.3390/plants15213354

**Published:** 2026-10-29

**Authors:** Feng Liu, Chunyuan Wu, Xiongchang Ou, Sisi Zhou, Lei Ren, Quansheng Yao, Rulin Zhan

**Affiliations:** 1 South Subtropical Crops Research Institute, Chinese Academy of Tropical Agricultural Sciences, Key Laboratory of Postharvest Physiology and Technology of Tropical Horticultural Products of Hainan Province, Zhanjiang 524091, China; 2 College of Coastal Agricultural Sciences, Guangdong Ocean University, Zhanjiang 524088, China; 3 Institute of Tropical Crops Genetic Resources, Chinese Academy of Tropical Agricultural Sciences, Sanya 572025, China

**Keywords:** mango, bacterial angular spot disease, SNP, KASP marker, disease resistance

## Abstract

Bacterial angular leaf spot, caused by *Xanthomonas citri* pv. *mangiferaeindicae* (Xcm), is an important disease that constrains mango production. Resistance-associated molecular markers can improve the efficiency of resistant germplasm screening and accelerate mango breeding; however, such markers remain limited for bacterial angular leaf spot. In this study, transcriptome and whole-genome resequencing data from the highly resistant cultivar ‘Renong No. 1’ and the highly susceptible cultivar ‘Keitt’ were reanalyzed to identify nonsynonymous single-nucleotide polymorphisms (SNPs) in candidate resistance-related genes. Among 86 candidate SNPs converted into Kompetitive Allele-Specific PCR (KASP) assays, 37 generated clear and stable genotype clusters in 25 mango accessions with known resistance phenotypes. SNP26 showed high consistency with the resistance phenotypes of these accessions and was subsequently used to genotype 322 mango germplasm accessions, which were separated into two genotype groups containing 34 and 288 accessions, respectively. Based on the SNP26 genotyping results, 72 accessions were selected for indoor fruit-puncture inoculation and classified into five resistance levels according to lesion development. SNP26 was located at position 1,299,510 on chromosome 15 within the coding region of *HSP83A*. The alleles were G in ‘Keitt’ and A in ‘Renong No. 1’, resulting in a leucine-to-valine substitution. Sanger sequencing confirmed the polymorphism at this locus. Following Xcm inoculation, *HSP83A* showed contrasting expression patterns between the two cultivars. Conserved domain and phylogenetic analyses indicated that *HSP83A* belongs to the HSP90/HtpG family and is closely related to homologous proteins in other plant species. These findings suggest that SNP26 is a promising resistance-associated marker for the preliminary identification of mango germplasm resistant to bacterial angular leaf spot and may facilitate marker-assisted selection in mango breeding.

## 1. Introduction

Mango (*Mangifera indica* L.) is one of the important tropical and subtropical fruits. It has a long history of cultivation in India and Southeast Asia [1]. It has been widely planted in many countries and regions worldwide [2]. In China, it is mainly cultivated in Hainan, Guangxi, Yunnan, Guangdong, and Sichuan [3]. In recent years, bacterial angular leaf spot has occurred in many mango-producing areas in China, posing a threat to mango production. Bacterial angular leaf spot is caused by *Xanthomonas citri* pv. *mangiferaeindicae* (Xcm) is one of the major diseases affecting mango production [4]. The disease can infect mango leaves, branches, and fruits. Black lesions with yellow halos appear after the leaves are damaged, and in severe cases, the leaves may fall off. Black-brown ulcers form after fruit damage, which seriously affects the appearance, quality, and yield of fruit [5,6]. There are differences in the sensitivity of mango organs and developmental stages to the disease, and fruit susceptibility usually increases with growth and development [7]. At present, the disease is mainly controlled through cultivation management and chemical agents, but these measures have drawbacks such as high labor requirements, inconsistent control efficacy, and potential environmental pollution [8,9]. Therefore, the cultivation of disease-resistant varieties is an important means of achieving continuous prevention and control of the disease.

The existing research on mango bacterial angular leaf spot mainly focuses on etiology, physiology, and disease control [10,11,12,13]; studies of resistance-related genes and molecular markers remain relatively limited. Previous studies have evaluated the resistance of different mango germplasms to bacterial angular leaf spot. For example, Mamta et al. [14] evaluated bacterial black spot incidence and severity in 20 mango cultivars, and Qi et al. [15] carried out resistance identification on 64 mango germplasms, which provided a phenotypic basis for disease-resistant germplasm screening and disease-resistant gene mining. However, mango is a perennial woody fruit tree with high genomic heterozygosity [16]. Many important traits are regulated by multiple minor genes, which increases the difficulty of mining resistance-related genes and loci [9]. At the same time, the disease phenotype may also be affected by the material development stage, inoculation conditions, and environmental factors. It is difficult to achieve rapid and stable screening of resistant materials only by phenotypic identification. Therefore, it is necessary to combine the resistance phenotype with genomic and transcriptome data to improve the reliability of resistance-related site screening. Jena and Chand [17] used multiple DNA marker systems, including SSR markers, to analyze genetic diversity among 70 Indian mango genotypes. In our previous study, Xu et al. [18] analyzed differential SNP loci between the highly resistant cultivar ‘Renong No. 1’ and the highly susceptible cultivar ‘Keitt’ using whole-genome resequencing data, providing a basis for further development of molecular markers associated with mango disease resistance.

Molecular markers can directly detect DNA sequence differences between individuals. They have the characteristics of abundance, wide distribution, high stability, and low environmental impact. They have been widely used in germplasm resource identification, genetic map construction, gene mapping, and molecular marker-assisted breeding [19,20,21]. However, many disease resistance traits are controlled by multiple genes or loci, and the development of molecular markers still needs to be integrated with stable resistance phenotypes. Single-nucleotide polymorphisms (SNPs) are widely distributed, abundant, and genetically stable in the genome, and are suitable for automated and high-throughput genotyping. They have become one of the most widely used molecular marker types in plant genetic research and molecular breeding [22]. By comparing sequencing data from resistant and susceptible materials with the reference genome, differential SNPs between varieties can be identified, and allele-specific markers can be developed based on the flanking sequence at the site, thereby improving the accuracy and efficiency of screening for resistant material [23]. Resistance-related SNP markers have been used for resistance-site screening, germplasm genotyping, and fine mapping of resistance genes [24,25,26,27]. In recent years, the combined analysis of high-density SNP typing and resistance phenotypes has been used to mine disease resistance-related genetic loci in various crops. For example, Cuenca et al. [28] fine-mapped the citrus Alternaria brown spot resistance locus to a 366 kb region, identified candidate resistance genes, and developed SNP markers for marker-assisted selection. Ndlovu et al. [29] combined genome-wide association analysis and linkage analysis to identify multiple marker–trait associations and candidate genes associated with northern leaf blight resistance in tropical maize. Khan et al. [30] identified stable genetic loci and candidate genes related to wheat rust resistance through multi-environment resistance phenotype identification and high-density SNP typing. These studies have shown that combining high-density genotyping, candidate gene analysis, and resistance phenotype identification is an important approach for exploring the genetic variation underlying crop disease resistance.

Kompetitive allele-specific PCR (KASP) is a fluorescent signal-based SNP genotyping technique [31]. The technique uses two allele-specific forward primers and a common reverse primer to distinguish biallelic variations by different fluorescence signals [32,33]. With the characteristics of simple operation, relatively low cost, high accuracy, and suitability for large-scale sample detection, it has been widely used in marker-assisted selection, genotyping, variety identification, and allele mining [32,34,35]. KASP technology can also be used to verify candidate SNPs identified by sequencing or gene mapping in a large population and to quickly screen the target alleles in the breeding population [36,37]. At present, disease resistance-related KASP markers have been applied to wheat [38], rice [39], and tomato [40]. They have been used to detect disease resistance alleles or candidate resistance loci in crops such as pepper [41], wheat [42], and grape [43]. However, research on KASP markers associated with disease resistance in fruit trees remains relatively limited, and the development and large-scale germplasm verification of KASP markers for bacterial angular leaf spot resistance in mango have received little systematic study.

Heat shock protein (HSP) is a class of highly conserved stress-responsive proteins widely found in prokaryotes and eukaryotes. Based on molecular weight and sequence characteristics, it can be divided into HSP90, HSP100, HSP70, HSP60, and the small-molecule HSP family [44]. HSP90 family proteins are a class of conserved ATP-dependent molecular chaperones that play important roles in protein folding, stabilization, activation, and assembly, and are involved in signal transduction, cell cycle regulation, and stress response [45]. In the plant immune response, HSP90 can form molecular chaperone complexes with proteins such as RAR1 and SGT1 and participate in the stability of immune receptors and the transmission of defense signals [46,47]. Recent studies have shown that HSP90 is involved in plant defense responses mediated by a variety of NLR immune receptors. During the interaction between cassava and Xanthomonas, HSP90 is regulated by the phosphorylation of host CPK1. It can be targeted by the pathogen effector protein XopC2, indicating that HSP90 is an important molecular node coordinating plant defense responses and pathogen infection [48,49]. Therefore, analyzing sequence variation and pathogen-induced expression characteristics of HSP90 family candidate genes in resistant and susceptible mango materials helps explore their potential relationship with resistance to mango bacterial angular leaf spot.

Based on the above research, this study used the highly resistant mango cultivar ‘Renong No. 1’ and the highly susceptible cultivar ‘Keitt’ as the main materials to re-analyze the transcriptome and whole-genome resequencing data obtained by the research group in the early stage. Candidate genes and non-synonymous SNPs potentially related to resistance were screened, and the candidate SNPs were converted into KASP markers. The candidate markers were preliminarily screened using 25 mango materials with known resistance phenotypes, and 322 mango germplasms were subsequently used to expand the genotyping population. At the same time, the key candidate sites were verified by Sanger sequencing, and the consistency between the SNP26 genotype and the resistance phenotype was evaluated by combining the results of indoor fruit-stabbing inoculations of 72 mango materials. The expression, response protein conserved domain, and phylogenetic relationship of the *HSP83A* gene after Xcm infection were analyzed. The purpose of this study was to develop candidate KASP markers related to mango bacterial angular leaf spot resistance, and to provide a technical basis for mango disease-resistant germplasm identification and molecular marker-assisted breeding.

## 2. Results

### 2.1. Candidate Genes and SNP Screening Related to Mango Bacterial Angular Leaf Spot Resistance

In this study, the number of SNP loci was counted in 10 transcriptome analysis groups (T1–T10) of ‘Renong No. 1’ and ‘Keitt’, and about 843,282 SNP loci were detected in each group. In addition, 751,006 transition sites and 462,106 transversion sites were recorded in the SNP variation types, with a total of 1,213,112 in the two types. On this basis, combined with the previous whole-genome resequencing data and the genotype differences, gene function annotation and transcriptome expression changes between the two varieties, 20 candidate genes that may be related to mango bacterial angular leaf spot resistance were screened (Table 1). The functional annotation of these genes involved plant–pathogen interaction, plant hormone signal transduction, MAPK signaling pathway, transcriptional regulation, and defense-related protein coding.

Among the above candidate genes, we further screened SNP loci that were homozygous in both varieties but with different genotypes and sequencing depth greater than 10, and preferentially selected non-synonymous mutations that may cause amino acid changes in the exon region. Finally, 86 candidate SNPs were obtained for subsequent KASP marker development.

### 2.2. Development and Preliminary Screening of KASP Markers for Candidate SNPs

#### 2.2.1. Development and Genotyping of KASP Markers for Candidate SNPs

**Figure 1:**
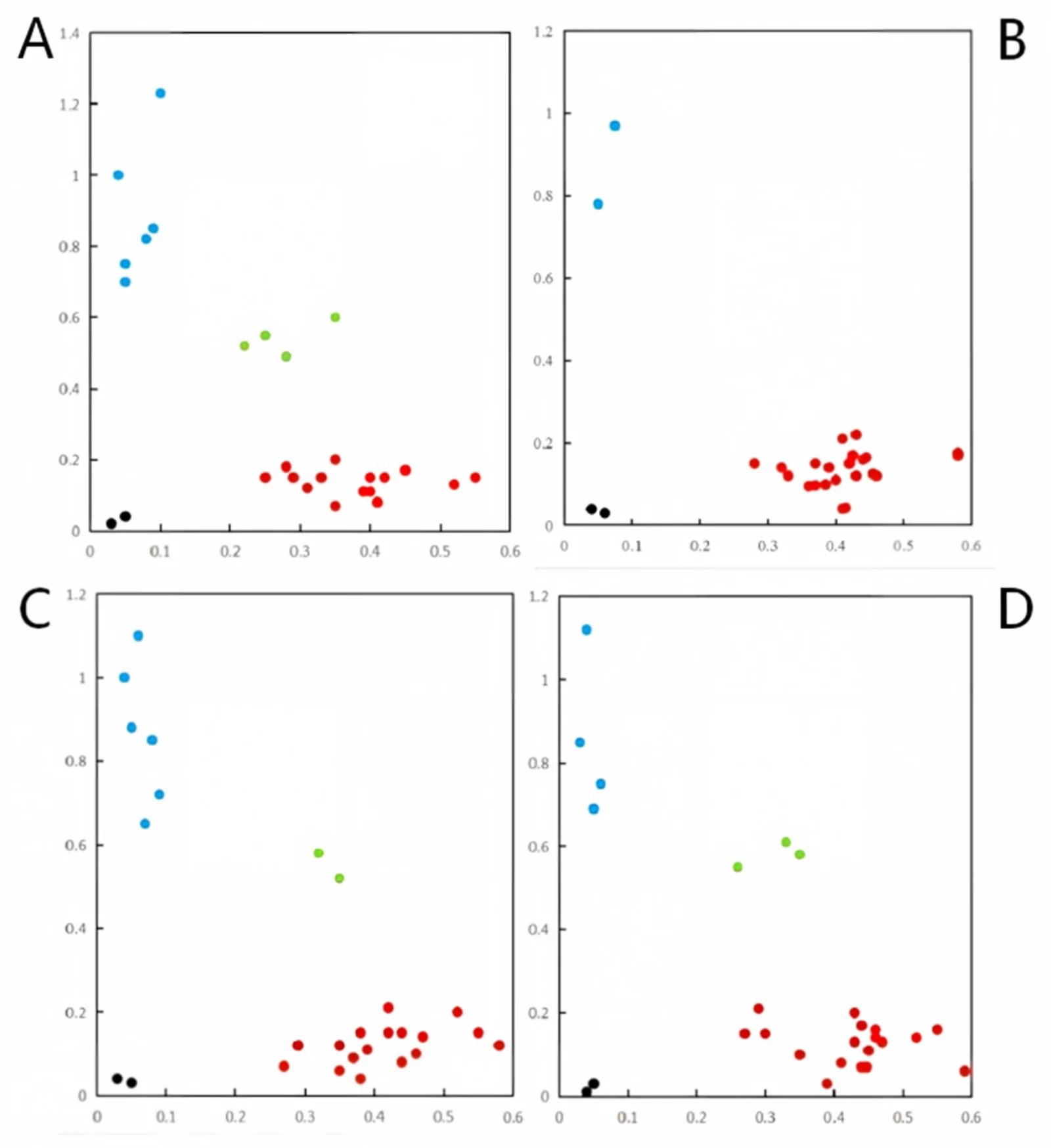
KASP genotyping results for representative candidate SNPs. Note: (**A**) SNP1: red dots represent the C/C genotype, blue dots represent the T/T genotype, and green dots represent the C/T heterozygous genotype. (**B**) SNP26: red dots represent the G/G genotype, and blue dots represent the A/A genotype. (**C**) SNP2: red dots represent the T/T genotype, blue dots represent the C/C genotype, and green dots represent the C/T heterozygous genotype. (**D**) SNP8: red dots represent the C/C genotype, blue dots represent the T/T genotype, and green dots represent the C/T heterozygous genotype. Black dots indicate no-template controls (NTCs).

Based on the 86 candidate SNPs and their flanking sequences screened in 2.1, KASP primers were designed, and the genotyping performance of the candidate markers was preliminarily verified using 25 mango materials numbered 1–25 in Appendix A. The results showed that 37 KASP markers produced clear and stable fluorescence signals, and the tested materials were divided into distinct genotype clusters, indicating that these markers had good genotyping performance and could be used for subsequent screening of resistance-related markers. The KASP typing results of some candidate markers are shown in Figure 1. All primer information is shown in Appendix B.

#### 2.2.2. Preliminary Screening of Resistance-Related KASP Markers

**Figure 2:**
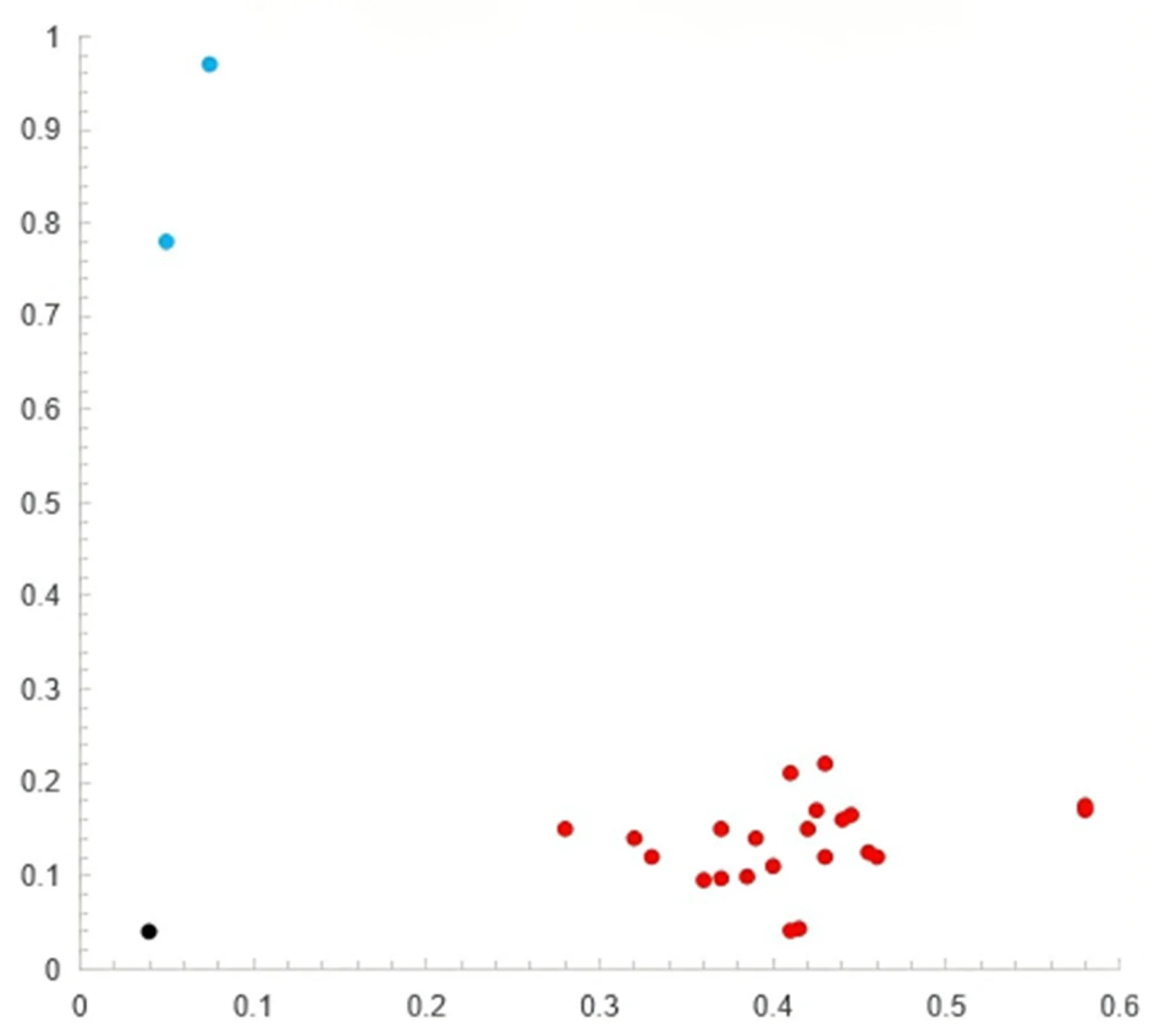
KASP genotyping results of 25 mango accessions using the SNP26 marker. Note: Red dots represent the G/G genotype, blue dots represent the A/A genotype, and black dots indicate no-template controls (NTCs).

Combined with the published results of bacterial angular leaf spot resistance identification of 25 mango materials, 37 KASP markers that can be stably genotyped were further screened. The 25 materials included one high-resistance material, one medium-resistance material, 10 medium-susceptible materials, nine susceptible materials, and four highly susceptible materials. The results showed that the 25 materials could be divided into two genotype groups by SNP26. One genotype group included one high-resistance and one medium-resistance material, and the other genotype group included 10 medium-susceptible, nine susceptible, and four highly susceptible materials. The genotype grouping was consistent with the published resistance-grade grouping (Figure 2).

Based on the resistance phenotype and KASP genotyping results from 25 materials, SNP26 was selected as a candidate resistance-related KASP marker for subsequent site-sequence verification and expanded-population genotyping.

#### 2.2.3. Genotyping of 322 Mango Germplasms by SNP26 Marker

**Figure 3:**
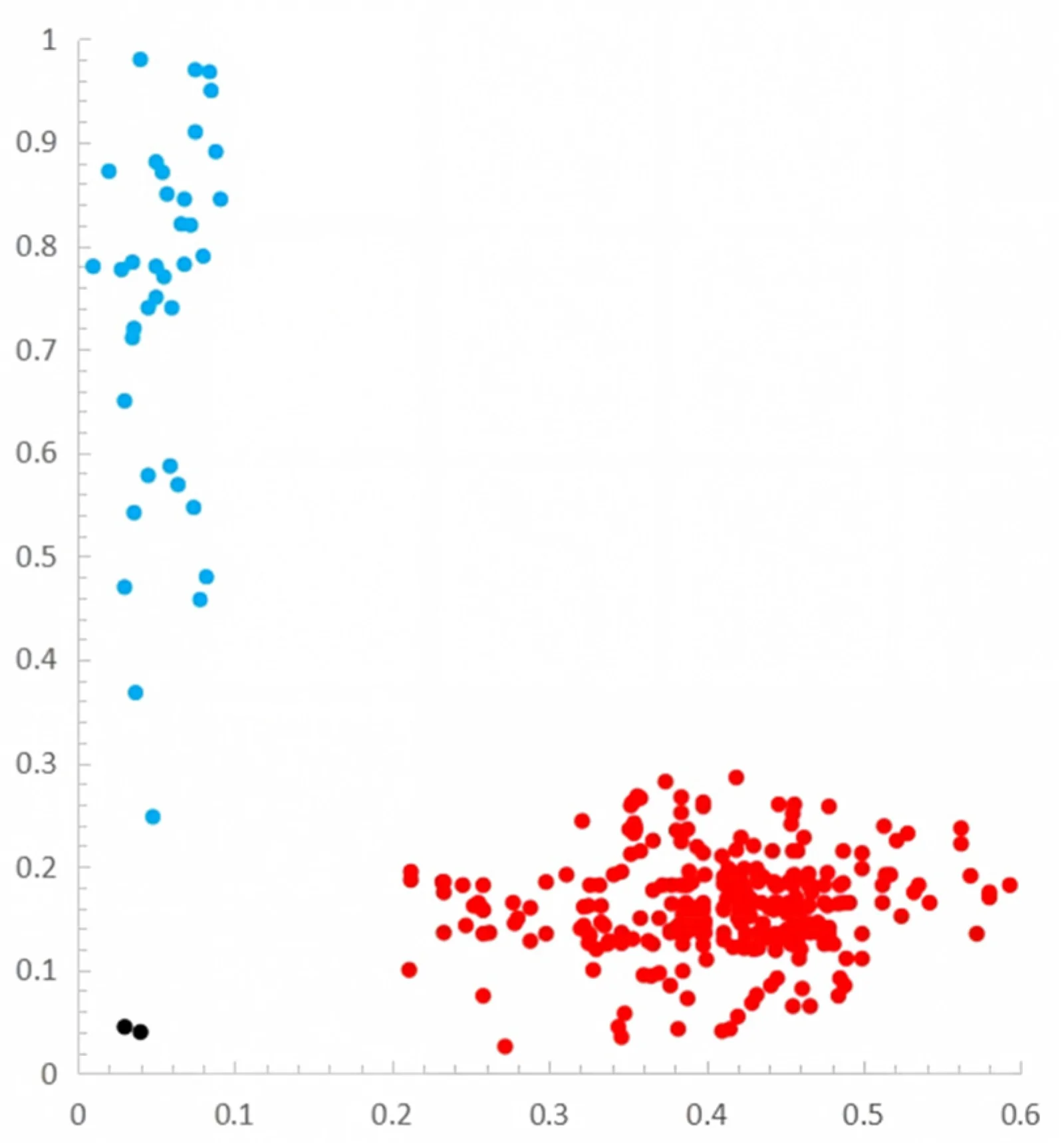
KASP genotyping results of 322 mango accessions using the SNP26 marker. Note: Red dots represent the G/G genotype, blue dots represent the A/A genotype, and black dots indicate no-template controls (NTCs).

In order to evaluate the genotyping effect of the SNP26 marker in the expanded mango germplasm population, 322 mango germplasm resources (Appendix A) were genotyped by KASP. The results showed that 322 materials could be clearly divided into two genotype groups by SNP26; one group included 34 materials, and the other group included 288 materials (Figure 3). The marker showed a clear and stable typing effect in the expanded population, which provided a basis for the subsequent evaluation of its application value in combination with the resistance phenotype.

#### 2.2.4. Validation of the SNP26 Polymorphism by Sanger Sequencing

Bioinformatics analysis showed that SNP26 was located at 1,299,510 bp on chromosome 15 of mango, within the coding region of the *HSP83A* (heat shock protein 83A) gene. This locus is guanine (G) in the highly susceptible variety ‘Keitt’, encoding leucine; in the highly resistant variety ‘Renong No. 1’, it is adenine (A), encoding valine, and the base difference leads to the substitution of leucine for valine.

**Figure 4:**
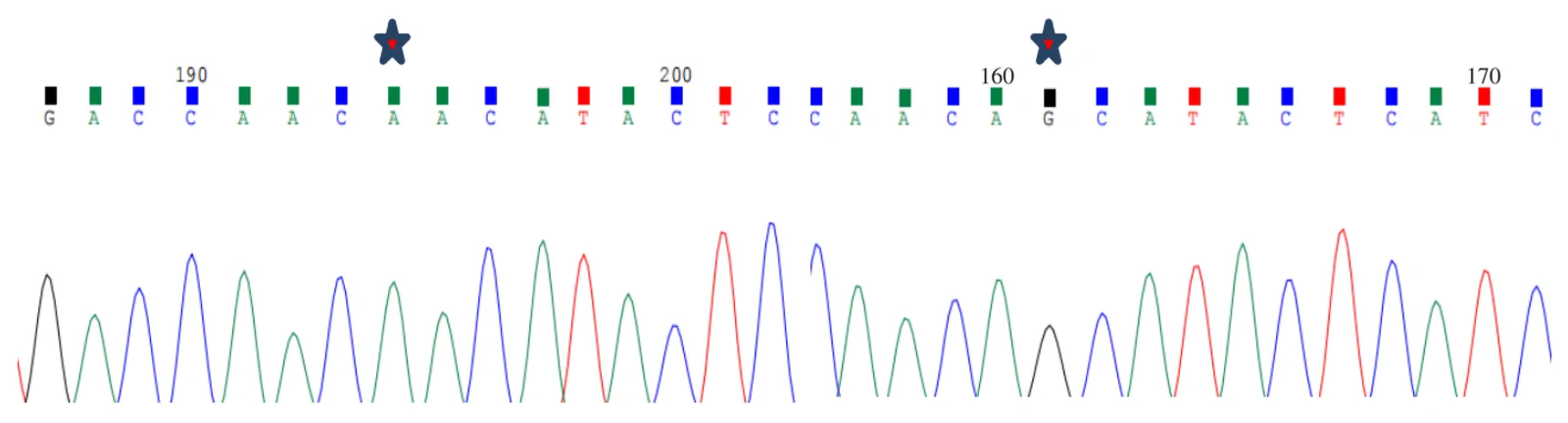
Sanger sequencing validation of the SNP26 locus in the *HSP83A* gene of ‘Keitt’ and ‘Renong No. 1’.Note: The stars indicate the SNP26 polymorphic site.

The cDNA of ‘Keitt’ and ‘Renong No. 1’ was used as a template to amplify the *HSP83A* gene fragment containing the SNP26 locus, and the amplified product was subjected to Sanger sequencing. Sequence alignment results showed that ‘Keitt’ was G at the SNP26 locus and ‘Renong No. 1’ was A, which was consistent with the results of whole-genome resequencing and verified the reliability of the SNP26 locus and its genotype difference (Figure 4).

### 2.3. Identification of Bacterial Angular Leaf Spot Resistance Phenotype of 72 Mango Germplasms

#### 2.3.1. Identification of the Resistance Level of 72 Mango Germplasms

A total of 72 mango accessions from the two SNP26 genotype groups were evaluated by indoor fruit puncture inoculation. Based on the lesion diameters at 9 days after inoculation, three accessions were classified as highly resistant (grade 1), seven as moderately resistant (grade 2), 41 as moderately susceptible (grade 3), 16 as susceptible (grade 4), and five as highly susceptible (grade 5). The 10 accessions with the A/A genotype comprised the three highly resistant and seven moderately resistant accessions. The 62 accessions with the G/G genotype comprised the 41 moderately susceptible, 16 susceptible, and five highly susceptible accessions. When grades 1–2 were grouped as resistant, and grades 3–5 as susceptible, the SNP26 genotype was significantly associated with observed resistance status in this panel (two-sided Fisher’s exact test, *p* < 0.001). Accession-level SNP26 genotypes, observed resistance grades, and the corresponding lesion-diameter classification ranges are provided in Appendix C.

#### 2.3.2. Symptoms of Mango Germplasms with Different Resistance Levels

**Figure 5:**
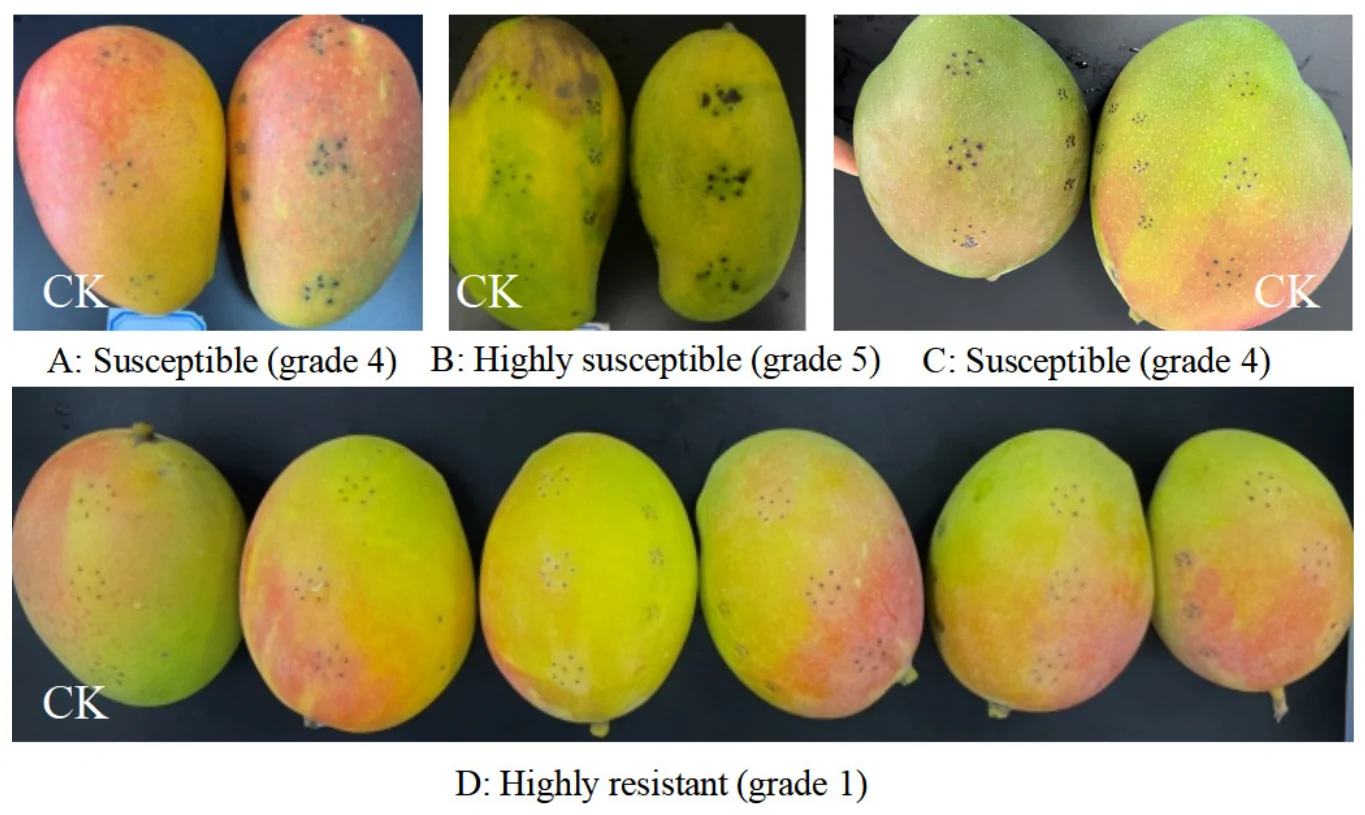
Representative symptoms of mango accessions with different resistance levels following Xcm inoculation. Note: (**A**) ‘Jingdongyinwanshu’, susceptible (grade 4); (**B**) ‘Keitt’, highly susceptible (grade 5); (**C**) ‘Guire No. 3’, susceptible (grade 4); (**D**) ‘Renong No. 1’, highly resistant (grade 1). CK indicates control fruits inoculated with sterile LB broth; all other fruits were inoculated with an Xcm suspension.

On the 9th day after inoculation, there were significant differences in the degree of lesion expansion among mango germplasms with different resistance levels. The lesion of the grade 1 material expanded slowly; the lesion diameter was small, and there was almost no significant change (Figure 5D); the lesion at the inoculation site of the grade 2 material expanded slightly; the lesion expansion degree of the grade 3 materials was higher than that of the grade 2 materials. The lesion expansion in grade 4 materials was more pronounced, and ulceration symptoms appeared. The lesion expansion of grade 5 materials was the most severe, and the lesion site showed obvious protrusions, which were similar to the typical symptoms of bacterial angular leaf spot in the field (Figure 5A–C).

### 2.4. The Expression of HSP83A Gene in Response to Bacterial Angular Leaf Spot Infection

**Figure 6:**
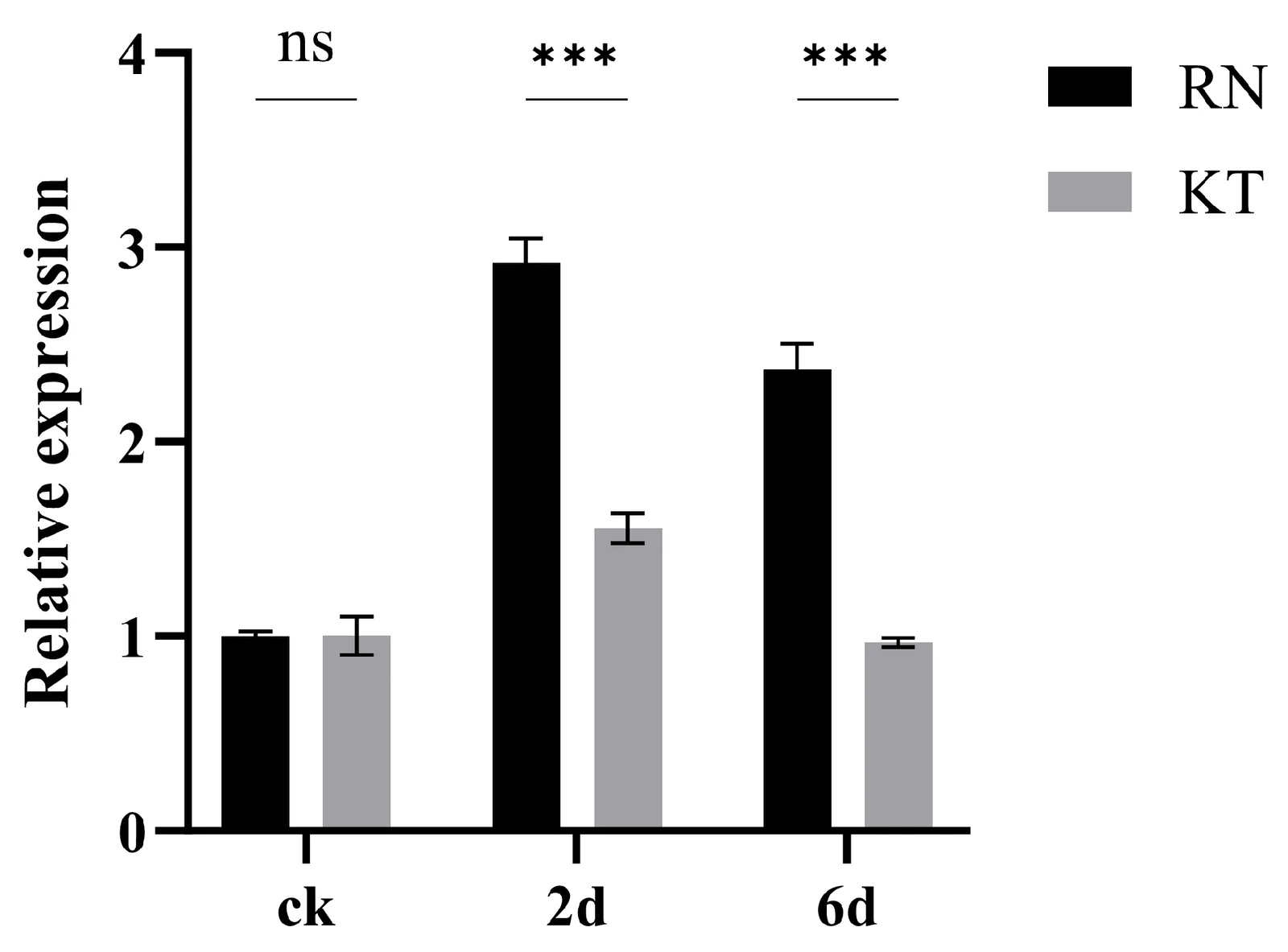
Relative expression levels of *HSP83A* in ‘Keitt’ and ‘Renong No. 1’ following Xcm inoculation. KT indicates ‘Keitt’; RN indicates ‘Renong No. 1’; CK indicates the control inoculated with sterile LB broth. *** indicates highly significant differences between RN and KT (*p* < 0.001); ns indicates no significant difference (*p* ≥ 0.05).

In order to analyze the expression response of *HSP83A* to Xcm inoculation, the highly resistant variety ‘Renong No. 1’ and the highly susceptible variety ‘Keitt’ were used as experimental materials. The relative expression levels of disease-resistant candidate genes in different time periods after inoculation in the two varieties were detected by real-time quantitative fluorescence PCR. The results showed that the relative expression of *HSP83A* in the two varieties increased first and then decreased, reaching the highest value at 2 days after inoculation, then decreased at 6 days. Based on the 0 d samples of each variety, the relative expression multiples of ‘Renong No. 1’ on 2 d and 6 d were higher than those of ‘Keitt’. At 6 d, the relative expression level of ‘Renong No. 1’ was still higher than its baseline level, while ‘Keitt’ was close to the baseline level (Figure 6). These results indicated that *HSP83A* showed different expression changes in the two varieties, suggesting that the gene may be involved in the response of mango to bacterial angular leaf spot pathogen inoculation.

### 2.5. Conserved Domain Features and Phylogenetic Relationships of the HSP83A Protein

**Figure 7:**
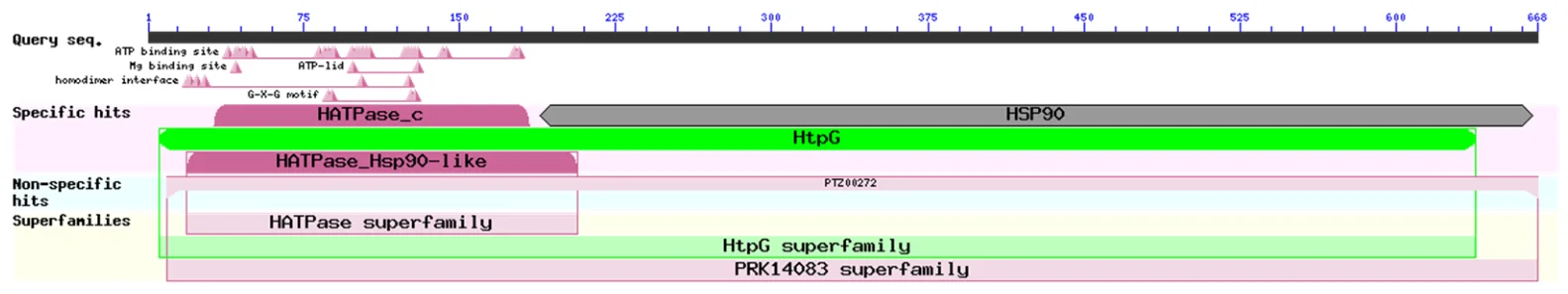
Conserved domain analysis of the mango HSP83A protein.

The conserved domain of the mango HSP83A protein was analyzed by NCBI Conserved Domain Search. The results showed that the HSP83A protein contained an HtpG-conserved domain in the 6–638 amino acid region and belonged to the HtpG superfamily (Figure 7).

**Figure 8:**
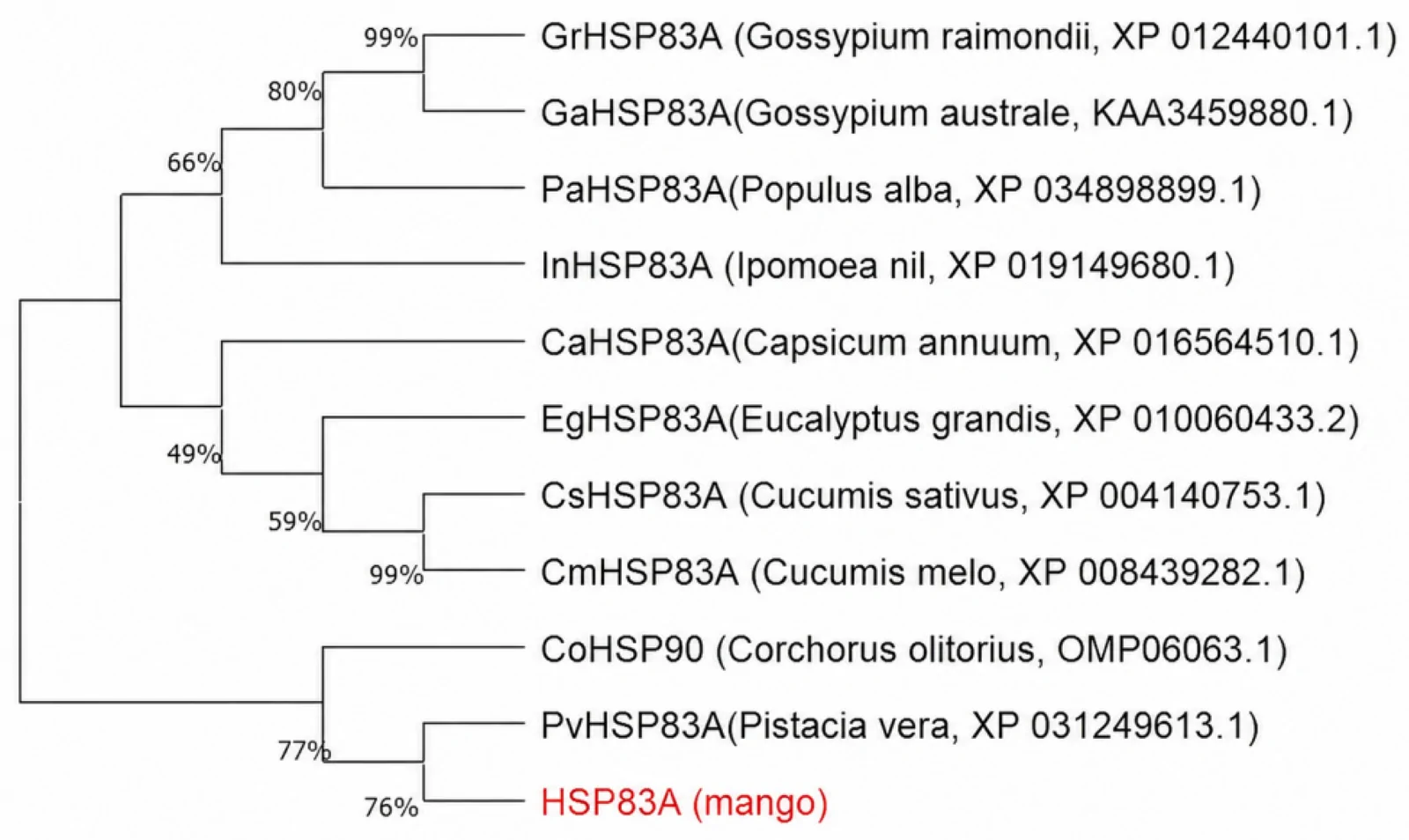
Phylogenetic relationships of HSP83A proteins from mango and other plant species. Red text indicates the mango HSP83A protein sequence analyzed in this study.

In order to analyze the phylogenetic relationship between mango HSP83A and other plant homologous proteins, 10 HSP83A homologous protein sequences from other plants were screened by NCBI BLASTP, and the phylogenetic tree was constructed with mango HSP83A. The results showed that mango HSP83A and Pistacia vera HSP83A were clustered into the same branch. The bootstrap support rate for this node was 76%, indicating a close phylogenetic relationship among the analyzed proteins (Figure 8).

## 3. Discussion

### 3.1. Screening of Resistance-Related SNPs and Development of KASP Markers

Bacterial angular leaf spot caused by Xcm is one of the main diseases affecting mango production. Existing research mainly focuses on etiology, physiology, and disease control, with few studies on disease resistance genes and molecular markers. Therefore, it is of great significance for disease-resistant germplasm screening and disease-resistant breeding to explore the genetic variation underlying resistance to mango bacterial angular leaf spot and to develop practical molecular markers.

Previous studies have used a combination of genomic variation analysis and resistance phenotype identification to screen crop disease resistance-related loci. For example, Ndlovu et al. [29] identified several marker–trait associations and candidate genes associated with leaf blight resistance in tropical maize through joint linkage and genome-wide association analyses. Khan et al. [30] identified stable genetic loci and candidate genes associated with rust resistance in wheat by combining multiple environmental resistance phenotypes with high-density SNP typing. These studies have shown that combining genomic variation, population genotyping, and resistance phenotypes is an effective way to screen candidate sites for disease resistance. At the same time, the candidate loci obtained by different techniques have different emphases. The population association analysis may be affected by population structure and phenotypic accuracy, and the single transcriptome analysis is also susceptible to limitations in tissue sampling and sampling time. Therefore, the combined analysis of multiple datasets helps narrow the range of candidate loci and improve the pertinence of candidate-loci screening.

In this study, combined with the transcriptome and whole-genome resequencing data of the highly resistant variety ‘Renong No. 1’ and the highly susceptible variety ‘Keitt’, and with gene function annotation and expression differences, priority was given to screening non-synonymous SNPs located in the exon region of disease resistance-related genes, which could cause amino acid changes and showed different homozygous genotypes in the two varieties; finally, 86 candidate SNPs were obtained. Among the KASP markers developed for these loci, 37 could form clear and stable genotype clusters. The above results indicate that the combined analysis of transcriptome and whole-genome resequencing data can effectively narrow the range of candidate sites and provide a technical basis for further screening of mango bacterial angular leaf spot resistance-related markers.

### 3.2. Relationship Between SNP26 Genotype and Resistance Phenotype

Candidate KASP markers need to be verified by genotyping and by assessing resistance phenotypes in materials with different genetic backgrounds to evaluate their application potential in disease-resistant germplasm screening. Holdsworth and Mazourek [41] developed KASP markers based on sequence variations in the coding regions of *pvr1* and *Bs3* resistance genes in pepper to distinguish resistant and susceptible alleles. Singh et al. [42] verified the application value of KASP markers for wheat disease resistance using QTL interval genotyping and sequencing. Wairich et al. [43] developed KASP markers for identifying downy mildew resistance in the candidate genes of the grape *Rpv3* resistance loci. These studies indicate that comparing KASP genotyping results with the material’s resistance phenotype is an important part of screening candidate markers for breeding applications.

In this study, 37 effective KASP markers were initially screened using 25 mango materials with known resistance phenotypes. The results showed that SNP26 could classify two disease-resistant materials and 23 materials with different susceptibility levels into two main genotype groups, showing high genotype–phenotype consistency. SNP26 was further used to genotype 322 mango germplasms, and the tested materials were divided into two genotype groups containing 34 and 288 materials, respectively. Subsequently, 72 materials were selected for indoor fruit puncture inoculation and resistance grade evaluation, thereby further expanding the range of materials for resistance phenotype evaluation. Based on the preliminary screening of 25 materials, the expanded population typing of 322 germplasms, and the resistance evaluation results of 72 materials, SNP26 has potential for preliminary screening of mango bacterial angular leaf spot-resistant germplasm. However, the resistance to this disease may be affected by multiple genes and environmental conditions. In the future, it is still necessary to further evaluate the accuracy and scope of application of this marker, in combination with resistance identification in larger populations and across multi-year and multi-site settings.

### 3.3. The Potential Role and Research Significance of HSP83A

The function of the candidate SNP gene and its response to pathogen infection can provide a biological basis for explaining the relationship between molecular markers and resistance phenotypes. In this study, SNP26 was located in the coding region of the *HSP83A* gene, and its base variation could change the corresponding amino acid, indicating that this site may be related to functional differences in HSP83A in addition to its genotyping value. Conserved domain analysis showed that the HSP83A protein contained a conserved domain of the HSP90/HtpG family. HSP90 is an ATP-dependent molecular chaperone that can form complexes with RAR1 and SGT1 and participate in immune receptor stability and plant defense signal transmission [46,48]. Recent studies have also shown that HSP90 is involved in the interaction between cassava and *Xanthomonas* [49], providing a theoretical basis for analyzing the potential disease resistance conferred by mango HSP83A.

This study found that SNP26 was G in the highly susceptible variety ‘Keitt’ and A in the highly resistant variety ‘Renong No. 1’, leading to the substitution of leucine for valine. The Sanger sequencing results were consistent with the whole-genome resequencing results, thereby confirming the authenticity of the base difference at this site. After Xcm infection, the relative expression of *HSP83A* in ‘Keitt’ increased at 2 d and decreased at 6 d after inoculation. At the same time, in ‘Renong No. 1’, it continued to increase with the prolongation of inoculation time and maintained a high level at 6 d, suggesting that *HSP83A* may be involved in the response of mango to Xcm infection. Phylogenetic analysis showed that mango HSP83A was closely related to Pistacia vera HSP83A. Based on the results of non-synonymous variation at SNP26, Sanger sequencing verification, pathogen-induced expression, and bioinformatics analysis, HSP83A may be involved in the response to mango bacterial angular leaf spot resistance, and it also provides a biological basis for SNP26 as a KASP marker related to resistance. Its specific role still needs to be further verified by genetic segregation, population co-segregation analysis, and gene function tests.

## 4. Materials and Methods

### 4.1. Plant Materials and Pathogens

#### 4.1.1. Plant Materials

In this study, the plant materials tested included the high-resistance mango variety ‘Renong No. 1’, the high-susceptibility variety ‘Keitt’, and 322 mango germplasm resources. ‘Renong No. 1’ and ‘Keitt’ were used for sequence verification of the SNP26 locus and *HSP83*A gene expression analysis. The 322 mango germplasms were preserved in the Mango Germplasm Resources Nursery of Huaping, Yunnan Province, and the Mango Germplasm Resources Nursery of the South Subtropical Crops Institute, Chinese Academy of Tropical Agricultural Sciences. The specific material names and numbers are shown in Appendix A. Among them, 25 materials numbered 1–25 in Appendix A were used for the preliminary screening of candidate KASP markers and the screening of resistance-related markers, and all 322 materials were used for the extended population genotyping of SNP26 markers.

Based on SNP26 genotyping results for 322 mango germplasms, 72 materials were selected from the two genotype groups for evaluation of bacterial angular leaf spot resistance. The materials used for indoor fruit-stabbing inoculation were about 70% mature, healthy, and free of obvious pests and diseases. The specific materials and their resistance levels are shown in Appendix C, and the representative fruit symptoms are shown in Figure 5. The young leaves used for genomic DNA extraction were collected from the above mango germplasm nursery. Healthy young leaves free of obvious pests and diseases were collected and returned to the laboratory for DNA extraction.

#### 4.1.2. Pathogens

The pathogen of mango bacterial angular leaf spot was isolated from mango fruit with typical symptoms of bacterial angular leaf spot in Huaping County, Yunnan Province. Through morphological identification and whole-genome sequence analysis, it was identified as Xcm. The strain was used for indoor fruit stab inoculation and *HSP83 A* gene expression analysis.

### 4.2. Screening of Disease-Resistant Candidate SNPs Based on Transcriptome and Whole Genome Resequencing Data

#### 4.2.1. SNP Identification and Screening

In this study, the transcriptome sequencing data of ‘Renong No. 1’ and ‘Keitt’ were analyzed, and the candidate sites were screened by combining the whole-genome resequencing data obtained in the previous study [18,50].

Transcriptome data were divided into 10 analysis groups according to variety, treatment, and sampling time, numbered T1-T10. T1 was the ‘Keitt’ 0 d untreated group; T2 and T4 were 2 d and 6 d treatment groups after Xcm inoculation of ‘Keitt’, respectively. T3 and T5 were the control groups of ‘Keitt’ inoculated with LB culture medium for 2 d and 6 d, respectively. T6 was the 0 d untreated group of ‘Renong No. 1’; T7 and T9 were the 2 d and 6 d treatment groups after Xcm inoculation of ‘Renong No. 1’, respectively. T8 and T10 were the control groups of ‘Renong No. 1’ inoculated with the LB culture medium for 2 d and 6 d, respectively.

The quality control of the original sequencing data of the transcriptome was carried out, and high-quality clean reads were obtained and aligned to the mango ‘Red Ivory’ reference genome. SNP detection was performed using GATK (4.4.0.0) software to filter low-quality sites and correct SNPs near InDel. The loci with reference allele support readings ≥ 2, variant allele support readings ≥ 3, and variant allele frequency of 0.1–0.9 were retained. On this basis, SNPs with a mutation quality score greater than 30, a sequencing depth of 10×–100×, and located in the exon region were further retained for subsequent candidate site screening.

The number of SNP loci detected in the above 10 analysis groups was counted, and the arithmetic mean was calculated. In addition, the number of transitions and transversions was counted according to the type of base substitution.

#### 4.2.2. Screening of Disease Resistance Related Candidate Genes and SNP

Combined with the transcriptome and whole-genome resequencing results of ‘Renong No. 1’ and ‘Keitt’, the Nr, EggNOG, GO, and KEGG databases were used to annotate the genes with differential variations between the two varieties. GO and KEGG enrichment analyses of differentially expressed genes were performed using a hypergeometric test. GO enrichment was based on a corrected *p*-value ≤ 0.05 as a significance criterion, and KEGG enrichment was based on a Q-value ≤ 0.05 as a significance criterion.

Comprehensive functional annotation, enrichment analysis results, and transcriptome expression information, focusing on genes that may be involved in plant–pathogen interaction, plant hormone signal transduction, as well as encoding transcription factors, kinases, peroxidases, and other defense-related proteins, were integrated. On this basis, combined with the gene expression level and the expression difference between the resistant and susceptible varieties, the candidate genes with high expression levels and different expression levels between the two varieties were preferentially screened.

SNP loci within the candidate genes were screened based on genotype, sequencing depth, genomic location, and functional effect. Loci with homozygous but different genotypes in ‘Renong No. 1’ and ‘Keitt’ and a sequencing depth greater than 10× in each sample were retained. Nonsynonymous SNPs in exonic regions were prioritized for subsequent KASP primer design.

### 4.3. KASP Marker Development and Genotyping

#### 4.3.1. Genomic DNA Extraction

Genomic DNA from mango leaves was extracted using the modified CTAB method [51]. The concentration and purity of DNA were determined using a spectrophotometer, and the integrity of DNA was assessed by 1% agarose gel electrophoresis. The concentration of qualified DNA samples was adjusted to 50 ng/μL for subsequent experiments.

#### 4.3.2. KASP Primer Design and Amplification

Based on the candidate SNPs and their flanking sequences obtained by 4.2 screening, KASP primers were designed using the SNPway online platform www.snpway.com (accessed on 24 September 2026). Two allele-specific forward primers and one common reverse primer were designed for each SNP locus. The 5 ′ ends of the two allele-specific forward primers were connected to the FAM fluorescent tag universal sequence (GAAGGTGACCAAGTTCATGCT) and the HEX fluorescent tag universal sequence (GAAGGTCGGAGTCAACGGATT), respectively. The length of the primers was 18–30 bp, and the length of the amplified fragment was less than 250 bp. KASP primers were synthesized by Bioengineering (Shanghai) Co., Ltd. The specific primer information is provided in Appendix B.

The total volume of the KASP reaction was 6 μL, comprising 1 μL of genomic DNA (50 ng/μL), 3 μL of 2× PARMS mix, 0.5 μL of primer mix, and 1.5 μL of ddH_2_O. The primer mix contained two allele-specific forward primers and one common reverse primer at a ratio of 1:1:2.

PCR Amplification Procedure: pre-denaturation at 94 °C for 15 min; 94 °C for 20 s, 65 °C for 1 min, each cycle annealing temperature decreased by 0.7 °C, a total of 10 cycles; then 94 °C for 20 s, 57 °C for 1 min, a total of 30–35 cycles; finally, it was kept at 35 °C for 1 min. After amplification, the FAM and HEX fluorescence signals were read using an Applied Biosystems QuantStudio 6 Flex Real-Time PCR System (Life Technologies Holdings Pte Ltd., Singapore), and the sample genotype was determined based on the fluorescence clustering results. Two to three no-template controls (NTCs) were set for each reaction plate.

#### 4.3.3. Preliminary Screening of KASP Candidate Markers

Twenty-five mango materials numbered 1–25 in Appendix A were selected to verify the genotyping effect of candidate KASP markers preliminarily. These 25 materials covered five resistance types: high resistance, medium resistance, medium susceptibility, susceptibility, and high susceptibility. The bacterial angular leaf spot resistance phenotype was derived from the published resistance identification results [15,52].

The DNA concentration was adjusted to 50 ng/μL, and KASP genotyping was performed according to the reaction system and amplification procedure described in Section 4.3.2. The typing performance of candidate markers was evaluated based on fluorescence signal intensity, the degree of separation between different genotype clusters, and typing stability. The genotyping results were compared with the existing resistance phenotypes of 25 materials to identify candidate KASP markers that show high consistency with bacterial angular leaf spot resistance and susceptibility phenotypes.

#### 4.3.4. Genotyping of 322 Mango Germplasms by SNP26 Markers

KASP genotyping was performed on all mango germplasms numbered 1–322 in Appendix A using the SNP26 marker obtained by screening. The concentration of each DNA sample was adjusted to 50 ng/μL. The KASP reaction system, PCR amplification procedure, and fluorescence signal reading method were the same as in Section 4.3.2. Each reaction plate was equipped with 1–3 NTCs. The SNP26 genotypes for each material were determined from the cluster formed by FAM and HEX fluorescence signals, and the number of distinct genotypes was counted.

#### 4.3.5. Sanger Sequence Verification of SNP26 Locus

The cDNA of ‘Keitt’ and ‘Renong No. 1’ were used as templates, and the *HSP83A* gene-specific primers containing SNP26 were used for PCR amplification. After the amplified products were detected by agarose gel electrophoresis, the gel containing the target band was cut, and the target fragment was recovered. The recovered PCR product was ligated into the *pEASY*-T1 Simple Cloning Vector and transformed into *Trans*1-T1 competent cells. Single colonies were picked and identified by colony PCR using M13 universal primers. The PCR products identified as positive were sent to Guangzhou Huada Gene Co., Ltd. for Sanger sequencing. The sequencing results of ‘Keitt’ and ‘Renong No. 1’ were compared with the SNP26 locus sequence obtained by the mango reference genome and whole-genome resequencing to verify the authenticity of the SNP26 locus G/A base difference.

### 4.4. Phenotypic Identification of Mango Bacterial Angular Leaf Spot Resistance

#### 4.4.1. Indoor Fruit Stabbing Inoculation

To further evaluate the correspondence between SNP26 genotypes and fruit resistance phenotypes, 72 accessions were randomly selected from the panel of 322 SNP26-genotyped mango germplasm accessions for indoor fruit puncture inoculation assays. Of the 72 selected accessions, 10 had the A/A genotype, and 62 had the G/G genotype. For each accession, 4 healthy fruits of similar size and approximately 70% maturity, without obvious signs of pests or diseases, were selected; 3 were used for Xcm inoculation, and 1 served as the control.

The fruits were soaked in a 1% NaClO solution for 1 min, rinsed 3 times with sterile water, and dried. The plum blossom needles, composed of 7 No. 3 insect needles (0.7 mm in diameter) with a diameter of about 1 cm, were used to stab three spots on each fruit surface. After Xcm was cultured in the LB medium for 2 days, 20 μL bacterial solution was added to each puncture site of the treated fruit, and an equal volume of sterile LB liquid medium was added to each puncture site of the control fruit.

The inoculated fruit was placed in a sterilized fruit tray and placed in a fresh-keeping box. The high-humidity environment was maintained by adding sterile water to a level below the fruit tray and placing wet cotton balls in the preservation box. The samples were then cultured under dark conditions at 28 °C and a relative humidity of about 100%.

#### 4.4.2. Disease Spot Measurement and Resistance Classification

Disease incidence and the extent of lesion expansion in the tested fruits were observed at 3, 6, and 9 days after inoculation. The lesion diameter (Φ) at the inoculation site was measured using the cross method and a vernier caliper [53], and the lesion diameter on the 9th day after inoculation was used to classify resistance. The resistance classification standard is as follows: 0.7 mm ≤ Φ ≤ 1.7 mm is grade 1 (highly resistant); 1.7 mm < Φ ≤ 2.7 mm is grade 2 (moderately resistant); 2. 7 mm < Φ ≤ 4. 7 mm is grade 3 (moderately susceptible); 4.7 mm < Φ ≤ 6.7 mm is grade 4 (susceptible); and Φ > 6.7 mm is grade 5 (highly susceptible).

### 4.5. Expression Analysis of HSP83A Gene Under Bacterial Angular Leaf Spot Infection

RNA Acquisition: The fruits of the highly susceptible variety ‘Keitt’ and highly resistant variety ‘Renong No. 1’ were used as experimental materials, and Xcm stab inoculation and LB liquid medium control treatment were carried out according to the method described in Section 4.4.1. The fruit tissues of the 0 d sample, 2 d and 6 d Xcm treatment groups, and LB control group were collected. Total RNA was extracted from each sample using the Quick RNA Isolation Kit (Huayueyang Biotechnology Co., Ltd., Beijing, China) according to the manufacturer’s instructions.

cDNA Synthesis: Total RNA extracted from mango was assessed using a microvolume UV spectrophotometer, and the optical density values ranged from 1.8 to 2.2. RNA integrity was assessed by 1.2% agarose gel electrophoresis, which showed intact RNA without obvious degradation. Genomic DNA removal and first-strand cDNA synthesis were performed using the FastKing RT Kit (With gDNase) (TIANGEN Biotech (Beijing) Co., Ltd., Beijing, China) according to the manufacturer’s instructions. The resulting cDNA served as the template for subsequent qRT-PCR analysis.

Primer Design: According to the CDS sequence of the *HSP83A* gene, Primer Premier 5.0 software was used to design specific qRT-PCR primers, and the mango *actin* gene was used as an internal reference. The forward primer 181-F of *HSP83A* was 5′-GCTGCTTGGTCACTGGCGAATA-3′, and the reverse primer 181-R was 5′-TTGTCATTCTTATCAGCCTCAGCCC-3′. The forward primer *Actin-F* of the internal reference gene was 5′-CCTTCAACTGACCAACAGGAGCAT-3′, and the reverse primer *Actin-R* was 5′-CTACATCACTGGTGAAAGTAAGAAA-3′.

qRT-PCR Reaction: qRT-PCR reaction was performed in 384-well plates. The total reaction volume was 10 μL, including 2 μL cDNA template, 1 μL corresponding primer mixture, 5 μL SYBR reaction solution, and 2 μL sterile water. The amplification procedure included pre-denaturation at 94 °C for 30 s. Then, denaturation at 94 °C for 5 s, annealing at 60 °C for 30 s, and extension at 72 °C for 10 s, giving a total of 40 cycles; subsequently, the melting curve analysis was performed as follows: 95 °C for 15 s, 65 °C for 1 min, and 95 °C for 15 s. Each sample was set up in 3 technical repetitions.

Relative Expression Calculation: Relative *HSP83A* expression was calculated using the 2^−ΔΔCt^ method, with *actin* as the reference gene. First, ΔCt was calculated for each replicate as ΔCt = Ct (target gene) – Ct (reference gene). The mean ΔCt of the day-0 samples from the corresponding cultivar was then used as the calibrator: ΔΔCt = ΔCt (test sample) − mean ΔCt (day-0 samples of the corresponding cultivar). Relative expression was calculated as 2^−ΔΔCt^ for each replicate, and results were expressed as the mean ± standard deviation. The Omicsmart cloud platform was used to analyze *HSP83A* expression data at different time points.

### 4.6. Conserved Domain and Phylogenetic Analysis of HSP83A Protein

The deduced amino acid sequence was obtained according to the coding sequence of the *HSP83A* gene, and the conserved domain of the HSP83A protein was analyzed by NCBI Conserved Domain Search (CD-Search). The amino acid sequence of the mango HSP83A protein was submitted to NCBI BLASTP for homology comparison, and the HSP83A protein sequence with high homology in other plants was screened.

The amino acid sequences of the HSP83A protein in mango and other plants were aligned using MEGA 7.0, and the phylogenetic tree was constructed by the neighbor-joining method. The bootstrap method was used to assess the reliability of each branch of the phylogenetic tree, with 1000 replicates.

### 4.7. Data Processing and Analysis

SNP26 genotypes were determined from the clusters formed by FAM and HEX fluorescence signals. Resistance grades were assigned based on lesion diameters at 9 days after inoculation, following the criteria described in Section 4.4.2. The number of accessions in each resistance grade was tabulated separately for each SNP26 genotype. For the association analysis, grades 1–2 (highly resistant and moderately resistant) were grouped as resistant, whereas grades 3–5 (moderately susceptible, susceptible, and highly susceptible) were grouped as susceptible. The association between SNP26 genotype and observed resistance status was evaluated using a two-sided Fisher’s exact test on a 2 × 2 contingency table, with each accession treated as one observation.

## 5. Conclusions

In this study, 86 candidate SNPs associated with mango resistance to bacterial angular leaf spot were identified, of which 37 were successfully converted into KASP markers with clear and stable genotyping clusters. Among these markers, SNP26, located at position 1,299,510 on chromosome 15, showed high consistency with the known resistance phenotypes of 25 mango accessions and divided 322 mango germplasm accessions into two distinct genotypic groups. Resistance evaluation of 72 mango accessions through artificial fruit inoculation revealed substantial variation in disease responses and classified the tested accessions into five resistance categories, providing additional phenotypic support for evaluating the SNP26 marker. Sanger sequencing further confirmed the A/G polymorphism between the resistant cultivar ‘Renong No. 1’ and the susceptible cultivar ‘Keitt’. SNP26 is located within the coding region of the *HSP83A* gene and results in an amino acid substitution. These findings indicate that SNP26 is a promising resistance-associated marker for the preliminary identification of mango germplasm resistant to bacterial angular leaf spot and may facilitate marker-assisted selection in mango breeding. Further validation in independent populations and different environments is required to confirm its predictive accuracy and broader applicability.

## Figures and Tables

**Table 1:** Candidate genes potentially associated with resistance to mango bacterial angular spot disease.

No.	Gene Symbol	Functional Annotation	SNP Type
1	*TIFY10A*	Plant hormone signal transduction	nonsynonymous SNV
2	*NPR1*	Plant hormone signal transduction	nonsynonymous SNV
3	*EIL1*	MAPK signaling pathway–plant	nonsynonymous SNV
4	*RPM1*	Disease resistance protein	nonsynonymous SNV
5	*PBS1*	Serine/threonine-protein kinase	nonsynonymous SNV
6	*NOA1*	Plant–pathogen interaction	nonsynonymous SNV
7	*RPS2*	Disease resistance protein	nonsynonymous SNV
8	*HSP83A*	Heat shock protein	nonsynonymous SNV
9	*CPK4*	Plant–pathogen interaction	nonsynonymous SNV
10	*chid1*	Chitinase	nonsynonymous SNV
11	*At1g64390*	Endoglucanase	nonsynonymous SNV
12	*At1g75680*	Endoglucanase	nonsynonymous SNV
13	*PTI1*	Plant–pathogen interaction	nonsynonymous SNV
14	*PAL6*	Phenylalanine ammonia-lyase	nonsynonymous SNV
15	*PAL*	Phenylalanine ammonia-lyase	nonsynonymous SNV
16	*RD19A*	Plant–pathogen interaction	nonsynonymous SNV
17	*CPK13*	Calcium-dependent protein kinase	nonsynonymous SNV
18	*GPX1*	Peroxidase	nonsynonymous SNV
19	*SGT1*	Plant–pathogen interaction	nonsynonymous SNV
20	*PRB1*	Pathogenesis-related protein 1-like	nonsynonymous SNV

## Data Availability

The RNA-sequencing data generated in this study have been deposited in the CNGB Sequence Archive (CNSA) of the China National GeneBank Database (CNGBdb) under accession number CNP0010084. https://db.cngb.org/data_resources/project/CNP0010084 (accessed on 24 September 2026). The other data supporting the findings of this study are available within the article and its appendices.

## Appendix A

**Table A1:** Information on 322 mango germplasm accessions.

ID	Variety	ID	Variety	ID	Variety	ID	Variety
1	Renong No. 1	87	Xinhong No. 2	173	Gaozhoulüsong	259	TA
2	Sijimang	88	Guire 80-17	174	Feilübin 12	260	524
3	Neelum	89	Jinxing	175	Meiguoheideng	261	Sannianmang
4	Huabianyu No. 3	90	Huabian (Yu) No. 1	176	Nandou No. 4	262	315Yugongci
5	Yuwen	91	Yunre 506	177	Nanxiu	263	Xingre 2
6	Kent	92	Haden	178	Dazhaihuangxin	264	Fu 1
7	Machesu	93	Huabian (Yu) No. 2	179	Hongguang No. 3	265	Fu 2
8	JinHuang	94	Xinshiji	180	Longxiang	266	1317 (Hong)
9	Magovar	95	Pan No. 1	181	Xiangya No. 22	267	Banmang
10	Renong No. 2	96	Hongguifei	182	Xiaoxiangya	268	1601
11	Yunre 507	97	Xiangjiao No. 1	183	krs	269	Baiyumang
12	Bandi	98	909	184	Dayuwen	270	Duopeizhong
13	Ganhong	99	Qinayia No. 1	185	Kaihao	271	1102
14	R2E2	100	Nanduomei	186	Moxige	272	AK
15	Guangxi 4	101	922	187	Miandianhongtao	273	Golek
16	Dongzhen Red Mango	102	918	188	Yuenanyinxigong	274	Thailand 504
17	Yuemang	103	920	189	Fu 3	275	20001
18	Haibao Mang	104	Apple	190	Mian No. 3	276	Guixiang (Guangxi No. 1)
19	Tainong	105	913	191	Dong	277	Mallika
20	Xingre 1	106	Yuexi No. 1	192	Taitai	278	zillate
21	Huabianyu No. 1	107	Datianxiang	193	Yuenanqing	279	20002
22	Shengxin	108	Zihua No. 3	194	Haibao	280	one
23	Red Ivory	109	Shengdelong	195	Jilaite	281	Pineapple Mango
24	Keitt	110	Nam Dok Mai	196	Xiamao	282	Small Philippine Mango (129)
25	Big White Jade	111	Hongwacheng	197	Fenhuang	283	20003
26	Sibenna	112	Aidehua	198	Repin 17	284	20004
27	Mian 8	113	Yongshengqishuxiangya	199	Repin 31	285	Thailand 506
28	Golek	114	Jingdongyinzaoshu	200	Hongping	286	Chuan Mang
29	Irwin	115	Panxihong	201	Bajisitan235	287	20007
30	Datou Mang	116	Yindu No. 2	202	901	288	lilley
31	Hongkeitt	117	Liuxiang	203	Yanzhi	289	Jinsui
32	Xiangjiao No. 2	118	Oct-97	204	Hubao	290	Sililanka 811
33	Mallika	119	Guo Xuehong orchard grafted accession	205	Wenxing	291	zill
34	Jingdongyinwanshu	120	Huabian (Yu) No. 5	206	Guire No. 278	292	Carabao
35	Guba No. 1	121	Huabian (Yu) No. 4	207	Guire No. 284	293	Lippens (97-11)
36	1034	122	Gui 10	208	Shitouzhaiyinhong	294	R2E2
37	Bangchpalli	123	Yu Anming orchard accession	209	Repin 19	295	Banganpalli
38	Pingguomang	124	Li Defu orchard accession	210	Mian No. 2	296	KRS
39	Magorar	125	Early Mango	211	Hongguang No. 1	297	Spooner
40	1506	126	Nov-97	212	Repin 30	298	Eduard
41	Geenn	127	97-15	213	Jinxing	299	9401
42	Guba No. 2	128	Guire No. 10	214	Hongping	300	Bambaroo
43	Baxihong	129	Lüsong	215	Mianer	301	Sargon
44	Guifei	130	Parrot mango	216	Guire 282	302	Vandyke
45	Alphonso	131	Kenxindun	217	Renong No. 10	303	Glenn
46	Huabian (Yu) No. 3	132	Haden No. 2	218	Taiya	304	Valencia Pride
47	Ru mang	133	Lüpimang	219	Mitao	305	Guifei 1
48	Gui 7	134	Guangxi No. 4	220	Anderson	306	20008
49	Li Dechun orchard accession	135	97-13	221	Jinlong	307	1030
50	Hutou mang	136	Yuanjiangxiangya	222	Tianyangxiang	308	kensinton
51	Lilley	137	Guangxi No. 5	223	Guire No. 2	309	814
52	504	138	Huangxianya	224	Tianyangtu	310	Dong Mang
53	Small Philippine Mango	139	May-97	225	Guire No. 4	311	Liuxiangmang
54	Apr-97	140	Laoyingzui	226	Guba	312	Qineiya No. 2
55	Thai Red	141	Bianliang No. 2	227	Xinhong	313	412
56	Caomei mang	142	Jinsui	228	Fengshunwuhe	314	135-1
57	97-18	143	Hong No. 11	229	Yexiang	315	Dasannian
58	Hong No. 8	144	Bianliang No. 1	230	Alphonso	316	509
59	Guire No. 82	145	EarlyGold	231	Xiangjiao mang	317	1002
60	Liuzhou Carabao	146	Maya	232	Xiaoqingpi	318	135-2
61	Jun-97	147	Anninghong	233	Thailand Carabao	319	703
62	506	148	Dec-97	234	Guba No. 3	320	1101
63	97-17	149	Baiyu	235	Guba No. 4	321	Thailand Sijimang
64	Chenpi	150	Jinbaihua	236	Thailand baihua	322	Hongjinhuang
65	Guangxi No. 8	151	Wacheng	237	Xiaoji		
66	Boluoxiang	152	97-2 (AX)	238	Xiaya		
67	Variant accession from Yang Chenghua orchard	153	Nang klangwan	239	Feizhoudaxiangya		
68	Shuiyingda	154	Tainong No. 1	240	Thailand yeshengmang		
69	Guire No. 3	155	Minzhutumala Liuxiang	241	Thailand No. 2		
70	Qingpi	156	Mengdi No. 1	242	Pairi		
71	Shanlin No. 1	157	Repin No. 6	243	Linsheng		
72	Liangre 2014-1	158	Long No. 12	244	Ai Mang		
73	Chenpixiang	159	Reyan No. 16	245	Gubasankexing		
74	Nüerhong	160	Bingxi No. 218	246	Siam Mango		
75	Self-bred accession (hybrid-derived variant)	161	Guire No. 71	247	Hongjinfeng		
76	Lingshuida	162	Cuiyun	248	Dayiwen		
77	Amou	163	Hong No. 10	249	yiwen		
78	Jinmixiang	164	95-111	250	Guifei 2		
79	Mashuai	165	Guire 108	251	Yiwenqingpi		
80	Guire No. 236	166	Liangre 2015-1	252	Hongjinlong		
81	Chuan	167	Guire 120	253	Naiyou		
82	pari	168	Longyanxiang	254	Guangxi No. Ba		
83	Huangyu	169	Sanyezihua	255	523		
84	Guixiang mang	170	Xishuangbannaxiaoxiangya	256	Fanghong		
85	Guire	171	Cuilü	257	Xingre 1		
86	Silika	172	Hongguang No. 6	258	chowsa		

## Appendix B

**Table A2:** KASP primer information for candidate SNPs.

Number	SNP Marker	Primer Sequence (5′-3′)	Type
1	SNP1	F:GAAGGTGACCAAGTTCATGCTGGCTTTTACTCCCGATTTGAACAC	C/T
		F:GAAGGTCGGAGTCAACGGATTGGCTTTTACTCCCGATTTGAACAT	
		R:TTTAAGCTGCTGAATTAGGCGTTC	
2	SNP2	F:GAAGGTGACCAAGTTCATGCTGCAGCTTAAACCTCCTGCAGT	T/C
		F:GAAGGTCGGAGTCAACGGATTGCAGCTTAAACCTCCTGCAGC	
		R:TGCACGTACACATCAATTTACCTG	
3	SNP3	F:GAAGGTGACCAAGTTCATGCTGCTTTGGGGAGTTCAGCCG	G/A
		F:GAAGGTCGGAGTCAACGGATTGCTTTGGGGAGTTCAGCCA	
		R:TCAAATCAAAACCCCCGAAAAGTT	
4	SNP4	F:GAAGGTGACCAAGTTCATGCTTTGTTAACTGGCAAGTTCGGCA	A/T
		F:GAAGGTCGGAGTCAACGGATTTTGTTAACTGGCAAGTTCGGCT	
		R:GTTATTGATGCTCAAACACAGCCT	
5	SNP5	F:GAAGGTGACCAAGTTCATGCTAAGACCCCCAGTATTCAAGCTTTT	T/G
		F:GAAGGTCGGAGTCAACGGATTAAGACCCCCAGTATTCAAGCTTTG	
		R:CGAATCACAGATGATGGGCAAAAA	
6	SNP6	F:GAAGGTGACCAAGTTCATGCTGGTGCGCTCTTTCATTCTGTAG	G/C
		F:GAAGGTCGGAGTCAACGGATTGGTGCGCTCTTTCATTCTGTAC	
		R:AAGGCAGACTCTCTCTCACTACTT	
7	SNP8	F:GAAGGTGACCAAGTTCATGCTTTCTAAAGAACTGCATTTGGCCGG	C/T
		F:GAAGGTCGGAGTCAACGGATTTTCTAAAGAACTGCATTTGGCCGA	
		R:ACCTTGGTCTTTCTTCCAGTTCTT	
8	SNP9	F:GAAGGTGACCAAGTTCATGCTGAGCCTATTCGCAGATCAGTCAGG	C/T
		F:GAAGGTCGGAGTCAACGGATTGAGCCTATTCGCAGATCAGTCAGA	
		R:GGCCAAATGCAGTTCTTTAGAAGT	
9	SNP12	F:GAAGGTGACCAAGTTCATGCTTCAAGAAGCTACCAATTTGGATTTCC	G/A
		F:GAAGGTCGGAGTCAACGGATTTCAAGAAGCTACCAATTTGGATTTCT	
		R:CCAACAAATTAGGCAGAGCTTGAA	
10	SNP14	F:GAAGGTGACCAAGTTCATGCTTCCCATACATCTCTATATACAAGATTAACC	C/T
		F:GAAGGTCGGAGTCAACGGATTTCCCATACATCTCTATATACAAGATTAACT	
		R:TGAAGCACTTAAGGAGCTCAAGAT	
11	SNP16	F:GAAGGTGACCAAGTTCATGCTGCATCTTGAGCTCCTTAAGTGCTT	T/A
		F:GAAGGTCGGAGTCAACGGATTGCATCTTGAGCTCCTTAAGTGCTA	
		R:CAGATTAACGATGAAGGACCTTGC	
12	SNP17	F:GAAGGTGACCAAGTTCATGCTCTCTGTTAAAGCCCCAAAGCCC	C/T
		F:GAAGGTCGGAGTCAACGGATTCTCTGTTAAAGCCCCAAAGCCT	
		R:CAGATTAACGATGAAGGACCTTGC	
13	SNP21	F:GAAGGTGACCAAGTTCATGCTTGCCACAACTGGAAAATTAACGATAA	A/G
		F:GAAGGTCGGAGTCAACGGATTTGCCACAACTGGAAAATTAACGATAG	
		R:GTAATCAGCTCCAACAATACCACG	
14	SNP22	F:GAAGGTGACCAAGTTCATGCTCCGGATCACCTTTGTCATCATGG	G/A
		F:GAAGGTCGGAGTCAACGGATTCCGGATCACCTTTGTCATCATGA	
		R:ATAATGCTTCGTTTCGCCTTTGAA	
15	SNP25	F:GAAGGTGACCAAGTTCATGCTCTGCAGGTACATCTCTGACACTT	T/A
		F:GAAGGTCGGAGTCAACGGATTCTGCAGGTACATCTCTGACACTA	
		R:GAAGTTGGCACGGATTTAATTCCA	
16	SNP26	F:GAAGGTGACCAAGTTCATGCTTGGATGCCATTGATGAGTATGCC	G/A
		F:GAAGGTCGGAGTCAACGGATTTGGATGCCATTGATGAGTATGCT	
		R:TCTTCATCAAGCTTTAGCCCTTCT	
17	SNP29	F:GAAGGTGACCAAGTTCATGCTTCTCCTTTTATTATTTCAAGTACGCTTT	T/C
		F:GAAGGTCGGAGTCAACGGATTTCTCCTTTTATTATTTCAAGTACGCTTC	
		R:TCCTCGTTGGTGTACATGAGAAAT	
18	SNP30	F:GAAGGTGACCAAGTTCATGCTTGATTATGAAGAAGAAACTGAGGGATAA	A/G
		F:GAAGGTCGGAGTCAACGGATTTGATTATGAAGAAGAAACTGAGGGATAG	
		R:CTTGCACTCTGCCAATATAAGCTC	
19	SNP34	F:GAAGGTGACCAAGTTCATGCTGATTTTGCAAAGCCTAGAAGTTGG	G/C
		F:GAAGGTCGGAGTCAACGGATTGATTTTGCAAAGCCTAGAAGTTGC	
		R:CTTCCTTCCTTGCTACTGTGTACT	
20	SNP36	F:GAAGGTGACCAAGTTCATGCTCCTTTGTAATCATGGAGGGATTGTAA	A/C
		F:GAAGGTCGGAGTCAACGGATTCCTTTGTAATCATGGAGGGATTGTAC	
		R:GGTCCCATTTAATCTCACTACTTGG	
21	SNP37	F:GAAGGTGACCAAGTTCATGCTCTGGTCTCTCCCAACAAGAATGG	G/T
		F:GAAGGTCGGAGTCAACGGATTCTGGTCTCTCCCAACAAGAATGT	
		R:AGAAAATGCAAACAAGGCTCTTGA	
22	SNP39	F:GAAGGTGACCAAGTTCATGCTTGATTATGTTTTGGGTGACAATCATAA	T/A
		F:GAAGGTCGGAGTCAACGGATTTGATTATGTTTTGGGTGACAATCATAT	
		R:CATAGCCCACAAGATAGCTCATCT	
23	SNP44	F:GAAGGTGACCAAGTTCATGCTACGCATCCAACAATAATGATCATTTTT	A/C
		F:GAAGGTCGGAGTCAACGGATTACGCATCCAACAATAATGATCATTTTG	
		R:TCATTTTTAACAGGTTGGAGTGGG	
24	SNP45	F:GAAGGTGACCAAGTTCATGCTTATCCTTGTAACAGGGTGTTGACC	C/T
		F:GAAGGTCGGAGTCAACGGATTTATCCTTGTAACAGGGTGTTGACT	
		R:ATGCTGGAATTTTTGGCAATGGTA	
25	SNP46	F:GAAGGTGACCAAGTTCATGCTCCAACATCGCCGCTCTTGTT	T/A
		F:GAAGGTCGGAGTCAACGGATTCCAACATCGCCGCTCTTGTA	
		R:ATGCTGGAATTTTTGGCAATGGTA	
26	SNP49	F:GAAGGTGACCAAGTTCATGCTACTTTGTAAAATTCCATCAGCTCC	C/T
		F:GAAGGTCGGAGTCAACGGATTACTTTGTAAAATTCCATCAGCTCT	
		R:GTGTATGCTGCCTCATGAAATCAA	
27	SNP51	F:GAAGGTGACCAAGTTCATGCTTGTTTTGATGCAAACATATTGGCTAA	A/C
		F:GAAGGTCGGAGTCAACGGATTTGTTTTGATGCAAACATATTGGCTAC	
		R:GTCTTCTTTATAACTTGGCCGACG	
28	SNP56	F:GAAGGTGACCAAGTTCATGCTACTTTTGTATTAATTTAGAATCATTCGACC	C/G
		F:GAAGGTCGGAGTCAACGGATTACTTTTGTATTAATTTAGAATCATTCGACG	
		R:GCGATTAAACCTCTGAGCTTCTTC	
29	SNP58	F:GAAGGTGACCAAGTTCATGCTAATTGATTATGGGGCGAACTATCC	C/G
		F:GAAGGTCGGAGTCAACGGATTAATTGATTATGGGGCGAACTATCG	
		R:CAAGTATGTCACACCGAATTCACC	
30	SNP60	F:GAAGGTGACCAAGTTCATGCTTAGAAGTCAACCAACCCTGAGAAA	T/C
		F:GAAGGTCGGAGTCAACGGATTTAGAAGTCAACCAACCCTGAGAAG	
		R:AGTCAAGGCTCAAGCAATTTTCAA	
31	SNP61	F:GAAGGTGACCAAGTTCATGCTCAGAACAAACAACGACACTGACC	C/G
		F:GAAGGTCGGAGTCAACGGATTCAGAACAAACAACGACACTGACG	
		R:TGATGACTCATCTGTTGACCCTTT	
32	SNP62	F:GAAGGTGACCAAGTTCATGCTGCTGTTGGAAAAATACTTTGCAGAA	A/G
		F:GAAGGTCGGAGTCAACGGATTGCTGTTGGAAAAATACTTTGCAGAG	
		R:GTTATTGTCCCACTGTTGTCTGTG	
33	SNP63	F:GAAGGTGACCAAGTTCATGCTGGGACAATAACACTTGAGGAACTTAAAA	A/T
		F:GAAGGTCGGAGTCAACGGATTGGGACAATAACACTTGAGGAACTTAAAT	
		R:TATAGTTACCGCATCCATAAGGGC	
34	SNP67	F:GAAGGTGACCAAGTTCATGCTGCAGTTCCAAAATTCGGTGCC	C/A
		F:GAAGGTCGGAGTCAACGGATTGCAGTTCCAAAATTCGGTGCA	
		R:TCTTGTCGCACTCTGTCAAAGATA	
35	SNP71	F:GAAGGTGACCAAGTTCATGCTAGTGGAGGAACTGAAGGGG	G/C
		F:GAAGGTCGGAGTCAACGGATTAGTGGAGGAACTGAAGGGC	
		R:CTTGCCCATTTTTAAGAACCCCAT	
36	SNP73	F:GAAGGTGACCAAGTTCATGCTCCAACAAAGAAGAAAGTTGAGAGAGG	G/T
		F:GAAGGTCGGAGTCAACGGATTCCAACAAAGAAGAAAGTTGAGAGAGT	
		R:TTGTGACTGCCCTTGTAAACAATC	
37	SNP75	F:GAAGGTGACCAAGTTCATGCTCTACCAATACCGCAACGTGACAA	A/G
		F:GAAGGTCGGAGTCAACGGATTCTACCAATACCGCAACGTGACAG	
		R:GCTCGAGTTCGTTGAAATTCATCA	

## Appendix C

**Table A3:** Resistance evaluation of 72 mango germplasm accessions to bacterial angular spot disease.

ID	Variety	SNP26 Genotype	Theoretical Resistance	Disease-Resistance Level	Lesion Diameter Range (mm)
1	Sibenna	Homozygous G/G	Susceptibility to disease	****	4.7 < Φ ≤ 6.7
2	Keitt	Homozygous G/G	Susceptibility to disease	*****	Φ > 6.7
3	Mian 8	Homozygous G/G	Susceptibility to disease	*****	Φ > 6.7
4	Yunre 507	Homozygous G/G	Susceptibility to disease	*****	Φ > 6.7
5	Golek Mang	Homozygous G/G	Susceptibility to disease	***	2.7 < Φ ≤ 4.7
6	JinHuang	Homozygous G/G	Susceptibility to disease	***	2.7 < Φ ≤ 4.7
7	Irwin	Homozygous G/G	Susceptibility to disease	***	2.7 < Φ ≤ 4.7
8	Datou Mang	Homozygous A/A	Disease resistance	**	1.7 < Φ ≤ 2.7
9	Shengxin	Homozygous G/G	Susceptibility to disease	****	4.7 < Φ ≤ 6.7
10	Red Ivory	Homozygous G/G	Susceptibility to disease	*****	Φ > 6.7
11	Hongkeitt	Homozygous G/G	Susceptibility to disease	***	2.7 < Φ ≤ 4.7
12	Renong No. 1	Homozygous A/A	Disease resistance	*	0.7 < Φ ≤ 1.7
13	Renong No. 2	Homozygous G/G	Susceptibility to disease	***	2.7 < Φ ≤ 4.7
14	Xiangjiao No. 2	Homozygous G/G	Susceptibility to disease	***	2.7 < Φ ≤ 4.7
15	Mallika	Homozygous G/G	Susceptibility to disease	****	4.7 < Φ ≤ 6.7
16	Jingdongyinwanshu	Homozygous G/G	Susceptibility to disease	****	4.7 < Φ ≤ 6.7
17	Guba No. 1	Homozygous A/A	Disease resistance	**	1.7 < Φ ≤ 2.7
18	01034	Homozygous G/G	Susceptibility to disease	***	2.7 < Φ ≤ 4.7
19	Bangchpalli	Homozygous G/G	Susceptibility to disease	***	2.7 < Φ ≤ 4.7
20	Apple	Homozygous A/A	Disease resistance	**	1.7 < Φ ≤ 2.7
21	Magorar	Homozygous G/G	Susceptibility to disease	***	2.7 < Φ ≤ 4.7
22	1506	Homozygous G/G	Susceptibility to disease	****	4.7 < Φ ≤ 6.7
23	Geenn	Homozygous G/G	Susceptibility to disease	***	2.7 < Φ ≤ 4.7
24	Guba No. 2	Homozygous G/G	Susceptibility to disease	***	2.7 < Φ ≤ 4.7
25	Kent	Homozygous G/G	Susceptibility to disease	***	2.7 < Φ ≤ 4.7
26	Baxihong	Homozygous A/A	Disease resistance	*	0.7 < Φ ≤ 1.7
27	Guifei	Homozygous G/G	Susceptibility to disease	***	2.7 < Φ ≤ 4.7
28	Alphonso	Homozygous G/G	Susceptibility to disease	****	4.7 < Φ ≤ 6.7
29	Huabian (Yu) No. 3	Homozygous G/G	Susceptibility to disease	***	2.7 < Φ ≤ 4.7
30	Ru mang	Homozygous G/G	Susceptibility to disease	***	2.7 < Φ ≤ 4.7
31	Gui 7	Homozygous G/G	Susceptibility to disease	***	2.7 < Φ ≤ 4.7
32	Big White Jade	Homozygous G/G	Susceptibility to disease	****	4.7 < Φ ≤ 6.7
33	Li Dechun orchard accession	Homozygous G/G	Susceptibility to disease	****	4.7 < Φ ≤ 6.7
34	Hutou mang	Homozygous G/G	Susceptibility to disease	***	2.7 < Φ ≤ 4.7
35	Lilley	Homozygous G/G	Susceptibility to disease	***	2.7 < Φ ≤ 4.7
36	504	Homozygous G/G	Susceptibility to disease	****	4.7 < Φ ≤ 6.7
37	Small Philippine Mango	Homozygous A/A	Disease resistance	**	1.7 < Φ ≤ 2.7
38	97-4	Homozygous A/A	Disease resistance	**	1.7 < Φ ≤ 2.7
39	Thai Red	Homozygous G/G	Susceptibility to disease	***	2.7 < Φ ≤ 4.7
40	Caomei mang	Homozygous G/G	Susceptibility to disease	***	2.7 < Φ ≤ 4.7
41	97-18	Homozygous G/G	Susceptibility to disease	***	2.7 < Φ ≤ 4.7
42	Hong No. 8	Homozygous G/G	Susceptibility to disease	****	4.7 < Φ ≤ 6.7
43	Guire No. 82	Homozygous G/G	Susceptibility to disease	***	2.7 < Φ ≤ 4.7
44	Liuzhou Carabao	Homozygous G/G	Susceptibility to disease	***	2.7 < Φ ≤ 4.7
45	97-6	Homozygous G/G	Susceptibility to disease	***	2.7 < Φ ≤ 4.7
46	506	Homozygous G/G	Susceptibility to disease	*****	Φ > 6.7
47	97-17	Homozygous G/G	Susceptibility to disease	***	2.7 < Φ ≤ 4.7
48	Chenpi	Homozygous A/A	Disease resistance	*	0.7 < Φ ≤ 1.7
49	Guangxi No. 8	Homozygous G/G	Susceptibility to disease	***	2.7 < Φ ≤ 4.7
50	Boluoxiang	Homozygous G/G	Susceptibility to disease	***	2.7 < Φ ≤ 4.7
51	Variant accession from Yang Chenghua orchard	Homozygous G/G	Susceptibility to disease	***	2.7 < Φ ≤ 4.7
52	Shuiyingda	Homozygous G/G	Susceptibility to disease	****	4.7 < Φ ≤ 6.7
53	Guire No. 3	Homozygous G/G	Susceptibility to disease	****	4.7 < Φ ≤ 6.7
54	Qingpi	Homozygous G/G	Susceptibility to disease	***	2.7 < Φ ≤ 4.7
55	Shanlin No. 1	Homozygous G/G	Susceptibility to disease	***	2.7 < Φ ≤ 4.7
56	Liangre 2014-1	Homozygous G/G	Susceptibility to disease	***	2.7 < Φ ≤ 4.7
57	Chenpixiang	Homozygous A/A	Disease resistance	**	1.7 < Φ ≤ 2.7
58	Nüerhong	Homozygous G/G	Susceptibility to disease	***	2.7 < Φ ≤ 4.7
59	Self-bred accession (hybrid-derived variant)	Homozygous G/G	Susceptibility to disease	***	2.7 < Φ ≤ 4.7
60	Lingshuida	Homozygous G/G	Susceptibility to disease	***	2.7 < Φ ≤ 4.7
61	Amou	Homozygous A/A	Disease resistance	**	1.7 < Φ ≤ 2.7
62	Jinmixiang	Homozygous G/G	Susceptibility to disease	****	4.7 < Φ ≤ 6.7
63	Mashuai	Homozygous G/G	Susceptibility to disease	***	2.7 < Φ ≤ 4.7
64	Guire No. 236	Homozygous G/G	Susceptibility to disease	***	2.7 < Φ ≤ 4.7
65	Chuan	Homozygous G/G	Susceptibility to disease	***	2.7 < Φ ≤ 4.7
66	pari	Homozygous G/G	Susceptibility to disease	***	2.7 < Φ ≤ 4.7
67	Huangyu	Homozygous G/G	Susceptibility to disease	****	4.7 < Φ ≤ 6.7
68	Guixiang mang	Homozygous G/G	Susceptibility to disease	****	4.7 < Φ ≤ 6.7
69	Guire	Homozygous G/G	Susceptibility to disease	***	2.7 < Φ ≤ 4.7
70	Silika	Homozygous G/G	Susceptibility to disease	****	4.7 < Φ ≤ 6.7
71	Xinhong No. 2	Homozygous G/G	Susceptibility to disease	***	2.7 < Φ ≤ 4.7
72	Guire 80-17	Homozygous G/G	Susceptibility to disease	***	2.7 < Φ ≤ 4.7

Note: (*) Highly resistant (HR); (**) moderately resistant (MR); (***) moderately susceptible (MS); (****) susceptible (S); (*****) highly susceptible (HS). Φ represents lesion diameter in mm. The listed ranges indicate the criteria used to classify resistance.
